# Genotyping of major histocompatibility complex Class II DRB gene in Rohilkhandi goats by polymerase chain reaction-restriction fragment length polymorphism and DNA sequencing

**DOI:** 10.14202/vetworld.2015.1183-1188

**Published:** 2015-10-09

**Authors:** Kush Shrivastava, Pushpendra Kumar, Nihar Ranjan Sahoo, Amod Kumar, Mohd. Faheem Khan, Amit Kumar, Arvind Prasad, B. H. M. Patel, A. Nasir, Bharat Bhushan, Deepak Sharma

**Affiliations:** 1 Division of Animal Genetics, Indian Veterinary Research Institute, Izatnagar, Bareilly, Uttar Pradesh, India; 2 Division of Parasitology, Indian Veterinary Research Institute, Izatnagar, Bareilly, Uttar Pradesh, India; 3 LPM Section, Indian Veterinary Research Institute, Izatnagar, Bareilly, Uttar Pradesh, India

**Keywords:** DRB1, major histocompatibility complex Class II, polymerase chain reaction-restriction fragment length polymorphism, Rohilkhandi goat, sequencing

## Abstract

**Aim:**

To study the major histocompatibility complex (MHC) Class II DRB1 gene polymorphism in Rohilkhandi goat using polymerase chain reaction-restriction fragment length polymorphism (PCR-RFLP) and nucleotide sequencing techniques.

**Materials and Methods:**

DNA was isolated from 127 Rohilkhandi goats maintained at sheep and goat farm, Indian Veterinary Research Institute, Izatnagar, Bareilly. A 284 bp fragment of exon 2 of DRB1 gene was amplified and digested using BsaI and TaqI restriction enzymes. Population genetic parameters were calculated using Popgene v 1.32 and SAS 9.0. The genotypes were then sequenced using Sanger dideoxy chain termination method and were compared with related breeds/species using MEGA 6.0 and Megalign (DNASTAR) software.

**Results:**

TaqI locus showed three and BsaI locus showed two genotypes. Both the loci were found to be in Hardy–Weinberg equilibrium (HWE), however, population genetic parameters suggest that heterozygosity is still maintained in the population at both loci. Percent diversity and divergence matrix, as well as phylogenetic analysis revealed that the MHC Class II DRB1 gene of Rohilkhandi goats was found to be in close cluster with Garole and Scottish blackface sheep breeds as compared to other goat breeds included in the sequence comparison.

**Conclusion:**

The PCR-RFLP patterns showed population to be in HWE and absence of one genotype at one locus (BsaI), both the loci showed excess of one or the other homozygote genotype, however, effective number of alleles showed that allelic diversity is present in the population. Sequence comparison of DRB1 gene of Rohilkhandi goat with other sheep and goat breed assigned Rohilkhandi goat in divergence with Jamanupari and Angora goats.

## Introduction

Goat is one of the major livestock species in Indian subcontinent and is important to the survival, economic, and social livelihood of millions of human being. India possesses rich goat population comprising 26.3% of total livestock of the country [1] and 14.6% of total world population of goat making it second largest goat population of the world [2]. Rohilkhandi goat is the small black colored goat of Rohilkhand region of northwestern Uttar Pradesh, which is a part of upper Ganges plains. It is a non-descript, local breed of goat and efforts to characterize it are underway, the breed is known locally for its hardiness, ability to withstand harsh climatic variations and is thought to be resistance to prevalent parasitic infestation.

Major histocompatibility complex (MHC) being the molecules of antigen recognition, have central role in disease resistance and susceptibility [3]. MHC in sheep and goats consists of three subgroups, MHC Class I, MHC Class II, and MHC Class III, among these the Class II molecule is divided into DQ and DR subtypes that are the most studied loci for disease resistance traits. The MHC Class II loci (both DQ and DR) exhibits high level of polymorphism in sheep and goat [4], among these two subgroups, the DRB locus is the most polymorphic and hence received the greatest attention of research groups for association studies in sheep [3]. The DRB locus forms the part of antigen binding groove in MHC cluster and hence exhibits high degree of variability, which enables it to identify a wide range of antigens and elicit proper immune response [5]. The high degree of variability at MHC loci is intended to be an outcome of balancing selection at this locus [6]. Due to this there is always a heterozygous excess at this loci so that the variability is maintained in varying degree of threat to body system, and hence there is always an expectation of population deviating from Hardy–Weinberg equilibrium (HWE) [7].

Because characterization of complete MHC genes is difficult due to its large size, therefore studies have been conducted fragment by fragment as per the importance of different regions. In sheep and goats, the polymorphism at DRB locus has been studied by a variety of methods including single-strand conformation polymorphism, restriction fragment length polymorphism (RFLP), cloning and sequencing [8], etc. Study of genetic polymorphism at MHC locus facilitates the identification of specific allelic variations that may be affecting disease resistance and susceptibility traits. Moreover, this may help in the development of markers for identification of resistance animals and assists in the development of breeding strategy for production of disease-resistant livestock [9]. The DRB fragment has been known to be associated with diseases conditions in sheep, and it has been used as a putative genetic marker in some sheep breeds for resistance/susceptibility, especially against gastrointestinal nematodes. The data on polymorphism in MHC Class II region is scanty in Indian goat breeds, especially not available in Rohilkhandi breed of goat. Therefore, because of the pronounced role of MHC Class II DRB region on disease susceptibility and resistance, the exon 2 of DRB1 gene was selected for the current study.

## Materials and Methods

### Ethical approval

Prior permission from Institutional Animal Ethical Committee (IAEC) was taken for collection of blood samples under sterile condition to carry out the current research work.

### Sample collection

A total of 127 blood samples were collected from a randomly mating population of Rohilkhandi goats maintained at sheep and goat farm, Indian Veterinary Research Institute (IVRI), Izatnagar, Bareilly. 5 ml whole blood was collected from jugular vein under sterile conditions in 2.7% ethylenediaminetetraacetic acid (EDTA) coated 15 ml polypropylene tubes and bought to lab on ice and stored at −21°C until further processing.

### Isolation of genomic DNA

Genomic DNA was isolated from whole blood using the method described by Sambrook *et al*. [10]. In brief, the blood was thawed and centrifuged at 3000 rpm for 20 min at room temperature to collect the white blood cell (WBC) pellet. The pellet was washed with chilled red blood cell lysis buffer (ammonium chloride 155 mM, potassium bicarbonate 9.9 mM and 29.9% 0.5 M EDTA) until it becomes free of red coloration. WBC pellet was then re-suspended in DNA extraction buffer (1% 1 M Tris pH 8.0; 8% 5 M NaCl; 0.4% 0.5 M EDTA) with subsequent addition of 10% sodium dodecyl sulfate at 200 µl per 10 ml of blood and finally adding proteinase K (20 mg/ml) in two pulse at 20 µl per 10 ml of blood with overnight incubation at 50°C. The contents were then centrifuged with tris-saturated phenol (pH 8.0) and aqueous upper phase was washed, once with phenol: chloroform:isoamyl alcohol (25:24:1) and chloroform: isoamyl alcohol (24:1). Finally, the DNA was precipitated with 10% of 3 M sodium acetate and equal amount of ice chilled isopropanol, washed twice with 70% ethanol air dried and resuspended in 0.3X Tris-EDTA buffer (pH 8.0).

### Amplification of MHC Class II DRB1 exon 2 region

A 284 bp fragment of exon 2 of DRB1 gene was amplified using a set of forward (5’-TATCCCGTCTCTGCAGCACATTTC-3’) and reverse (5’-TCGCCGCTGCACACTGAAACTCTC-3’) primers [11]. The 25 µl of polymerase chain reaction (PCR) reaction mixture was prepared using 10 pmoles of each primer, 12.5 µl of PCR master mix (Thermo scientific), 80-100 ng DNA template and nuclease free water to make volume. The amplification was carried out using a pre-programmed thermal cycler (ABI thermocycler) with the following conditions: Initial denaturation at 95°C for 5 min, followed by 39 cycles of denaturation at 94°C for 1 min, annealing at 59.0°C for 45 s and extension at 72°C for 1 min, and finally the last extension at 72°C for 5 min. The PCR products were checked by agarose gel electrophoresis using 1.0% agarose gel in 1× Tris-borate-EDTA buffer at 6 V/cm for 1 h.

### Restriction digestion

Restriction digestion of PCR products was carried out using two different restriction enzymes, *i.e*., TaqI and BsaI. Reaction conditions for restriction digestion were followed as per manufacturer’s instructions. In brief, 5-10 units restriction enzyme, 1X digestion buffer (supplied with the enzyme) and 15 µl amplified PCR product and the reaction volume was made to 20 µl by nuclease free water and kept for overnight digestion at 65°C for TaqI (Thermo Scientific) and 37°C for BsaI (New England Biolabs). The digested products were run on agarose gel from 2% to 4% as expected size of fragments with suitable DNA marker. Gel photos were recorded on Syngene gel doc system, and band sizing was done by Gel analyzer (freeware) 2010 and confirmed by semilog plotting using 100 bp size standard marker (Thermo scientific). The digested products were genotyped, and gene and genotype frequencies obtained were estimated using PROC ALLELE procedure of SAS. 9.0. Test for HWE and neutrality ratios was done using POP GENE v 1.32.

### Sanger sequencing of PCR products

The products obtained by PCR were then cleaned to remove salts and excess primer by GeneJet PCR purification kit. In brief, 25 µl of PCR product was mixed with equal amount of binding buffer, incubated for a minute and the mixture was then transferred to GeneJet purification column and centrifuged at 10,000 rpm for 1 min, 700 µl of wash buffer was then added on the column and centrifuged again at 10,000 rpm for 1 min (one more spinning of empty column may be required for removal of remaining wash buffer). The solution was finally eluted by adding 50 µl of elution buffer and collected in 1.5 ml microcentrifuge tube. The representative products obtained were then sequenced by Sanger’s dideoxy chain termination method (Eurofin Scientific) and the sequences so generated were subjected to BLAST (www.ncbi.nlm.nih.gov/BLAST) analysis to ascertain that sequences were of DRB gene and submitted to database. The corresponding nucleotide sequences from *Capra hircus* (AY496062 and JF416295) and *Ovis aries* (KC733432, KC733428, AB796330, FR751088, HE801965, and KM588654) were retrieved from NCBI database and were aligned using the ClustalW method of MEGA 6.0. Sequence comparison and phylogenetic tree were developed using MegAlign (DNASTAR).

## Results

### Population genetic analysis

**Figure-1:**
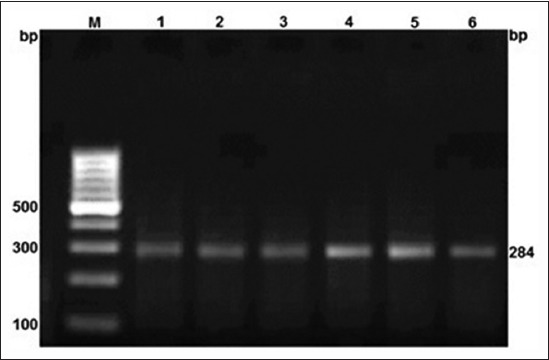
Amplified product of 284 bp of major histocompatibility complex Class II DRB1 in Rohilkhandi goat.

**Figure-2:**
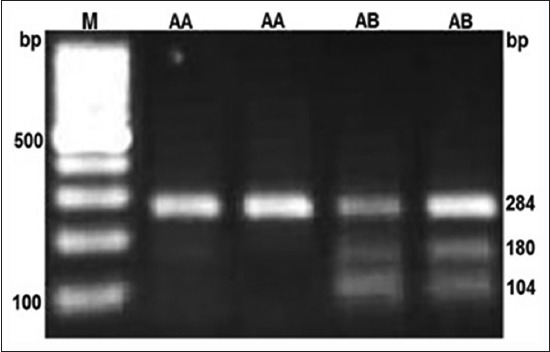
BsaI digestion of DRB1 gene in Rohilkhandi goats.

**Figure-3:**
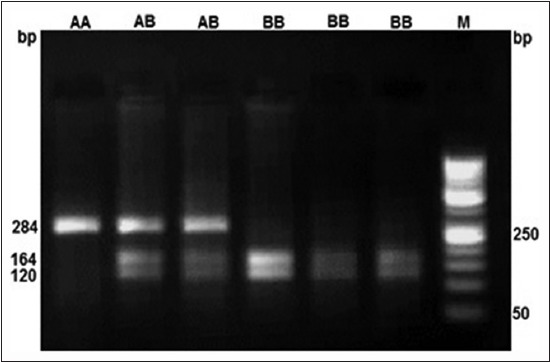
TaqI digestion of DRB1 gene in Rohilkhandi goats.

A 284 bp region of MHC Class II DRB 1 gene in Rohilkhandi goats was amplified (Figure-1). The digestion of PCR product by BsaI and TaqI restriction enzymes revealed AA, AB and AA, AB, BB genotypes, respecctively (Figures-2 and -3). The allelic and genotypic frequency at both marker loci (TaqI and BsaI) are shown in Table-1. The genotypic frequency of homozygotes, *i.e*., AA (0.764) and BB (0.622) was higher than heterozygotes for TaqI and BsaI loci, respectively. There was a deficiency of BB genotype at BsaI loci in the studied population (Table-1). The frequency of B allele (0.787) was higher at TaqI locus, whereas it was higher for A allele (0.882) at BsaI loci.

**Table-1:** Allele-wise gene and genotype frequency.

Allele frequency					Genotype frequency				
	
Locus	Allele	Count	Frequency	SE	Locus	Genotype	Count	Frequency	SE
TaqI	A	54	0.213	0.026	TaqI	AA	6	0.047	0.015
	B	200	0.787	0.026		AB	42	0.331	0.015
BsaI	A	224	0.882	0.019		BB	79	0.622	0.015
	B	30	0.118	0.019	BsaI	AA	97	0.764	0.005
	-	-	-	-		AB	30	0.236	0.005

Both the loci showed a polymorphism information content (PIC) value of 0.278-0.186 with heterozygosity values ranging from 0.331 to 0.236, for TaqI and BsaI, respectively. The summary of markers in relation to PIC, and test for HWE are given in Table-2. Further analysis of marker data was done by POP GENE v 1.32 to obtain other heterozygosity statistics. The observed number of alleles at both loci was two and the mean effective number of alleles was 1.383 and 0.440, respectively for TaqI and BsaI. The mean Shannon’s information index value was 0.440 (Table-3). As expected, the PIC and Shannon’s information index values were more in TaqI locus as compared to BsaI loci due complete absence of one genotype at BsaI. The heterozygosity statistics at both loci are given in detail in Table-3. The F_IS_ value [12] for both allele (A and B) at TaqI locus was 0.0122 and at BsaI locus was −0.133. The negative value denotes an excess of heterozygotes, however, at BsaI loci the negative value may also have occurred due to the absence of one genotype. Test for population in HWE showed population to be in equilibrium at both loci instead of absence of one genotype at BsaI locus.

**Table-2:** Chi-square test values for HWE for both loci.

Locus	Number of individuals	Number of alleles	PIC	Test for HWE			

				Chi-square	Df	Pr>Chi-square*	p** exact
TaqI	127	2	0.278	0.019	1	0.890	0.800
BsaI	127	2	0.186	2.278	1	0.131	0.211

* p value for the Chisquare test,

** an estimate of the exact p value for the HWE test. HWE=Hardy–Weinberg equilibrium, PIC=Polymorphism information content

**Table-3:** Heterozygosity statistics of both loci (TaqI and BsaI).

Locus	Sample size	Obs_Hom*	Obs_Het*	Exp_Hom^1^	Exp_Het^1^	Nei^2^	ne^3^	na^4^	I^5^
TaqI	254	0.669	0.331	0.664	0.336	0.335	1.503	2.000	0.517
BsaI	254	0.764	0.236	0.791	0.209	0.208	1.263	2.000	0.363
Mean		0.717	0.284	0.727	0.273	0.272	1.383	2.000	0.440
SD		0.067	0.067	0.090	0.090	0.089	0.169	0	0.109

* Observed homozygosity and heterozygosity,

1 Expected homozygosity and heterozygosity,

2 Nei’s expected heterozygosity,

3 ne=Effective number of alleles,

4 na=Observed number of alleles,

5 I=Shannon information index, SD=Standard deviation

### Nucleotide and amino acid sequence variability

**Figure-4:**
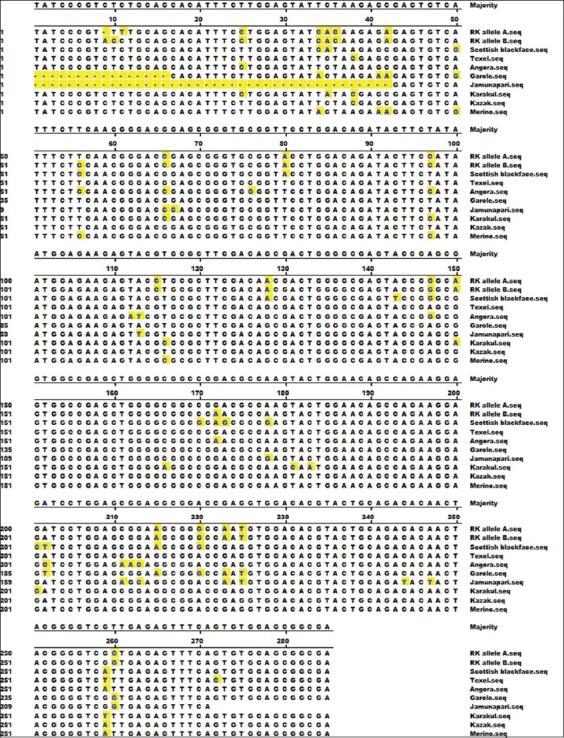
Nucleotide alignment report of analyzed sequences. RK allele A: Rohilkhandi goat allele A (Acc no. KP888556); RK allele B: Rohilkhandi goat allele B (Acc. no. KP888557).

The alleles (A and B) of amplified fragment of DRB1 gene were sequenced by Sanger’s dideoxy chain termination sequencing method [13] and were submitted to NCBI GenBank database with accession numbers KP888556 and KP888557. The two genotypes of the present study and the corresponding sequences of Jamunapari (JF416295) and Angora (AY496062) goats, Chinese merino (KC733428), Garole (KM588654), Texel (FR751088), Karakul (AB796330), Scottish Blackface (HE801965), and Kazak (KC733432) sheep breeds were aligned using MEGA 6.0 software. Nucleotide sequence variability was found at five places between the two alleles of Rohilkhandi goat *viz*. 9^th^, 10^th^, 11^th^, 56^th^, and 172^nd^ position. The nucleotide variability between Rohilkhandi goat and other breeds/species was found at total 46 places and is shown in Figure-4.

**Figure-5:**
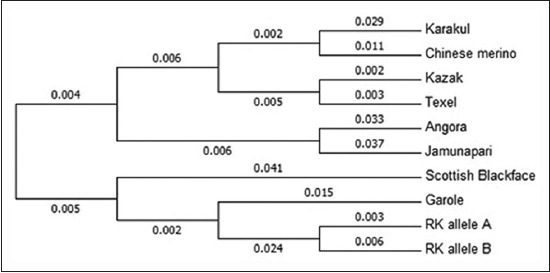
Neighbor-joining phylogenetic tree created between Rohilkhandi goat and other breed/species. RK allele A: Rohilkhandi goat allele A (Acc. no. KP888556); RK allele B: Rohilkhandi goat allele B (Acc. no. KP888557).

A phylogenetic neighbor-joining tree by p distance model with 1000 bootstrap replications was also created using all the sequences including the 1^st^, 2^nd^, and 3^rd^ codon positions plus non-coding regions with gaps eliminated. The phylogenetic analysis placed alleles of Rohilkhandi goat in one cluster and other the sheep and goat breeds showed scattered grouping (Figure-5).

**Figure-6:**
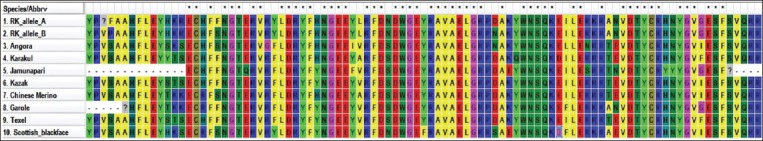
Amino acid alignment by Clustal W of the studied sequence (using MEGA 6.0). RK allele A: Rohilkhandi goat allele A (Acc. no. KP888556); RK allele B: Rohilkhandi goat allele B (Acc. no. KP888557).

The sequences were then translated, and the obtained amino acid sequences were aligned using MEGA 6.0. The amino acid alignment revealed three variable sites between the two alleles of Rohilkhandi goats including one stop codon (Figure-6).

## Discussion

The Rohilkhandi goat is a small sized native breed of areas of western Uttar Pradesh and is supposed to be resistant to common nematode infestation of goats. MHC Class II genes are central in conferring resistance/susceptibility to parasitic infestation [14]. Polymorphism at MHC loci is one of the major drivers of species survival. The polymorphism was reported in MHC Class II DRB1 gene of Rohilkhandi goats by PCR-RFLP and nucleotide sequencing. The polymorphism at this locus has been extensively studied by PCR-RFLP and is advocated to be used as genetic marker for nematode resistance/susceptibility [9]. The population genetic analysis of the genotypic data showed both loci to be in HWE with comparatively less heterozygosity, however, earlier PCR-RFLP studies on this gene have reported heterozygote excess and significant deviations from HWE using multiple restriction enzymes [9,15]. Previous reports have also shown that population that is relatively closed and breeding randomly within the herd tends to be in HWE [16]. The nucleotide comparison between Rohilkhandi goats and other sheep and goat breeds was done using Mega 6.0 and Megalign (DNASTAR) software which showed total of 46 points of mismatch at DNA sequence level between all the sequences. Construction of phylogenetic tree showed Rohilkhandi goat in a completely different cluster closer to Scottish blackface and Garole that are known to be gastrointestinal resistant [17,18].

## Conclusion

In the current study, the MHC Class II DRB1 gene of Rohilkhandi goats was studied by PCR-RFLP and nucleotide sequencing. The importance of this fragment is that it forms the part of antigen binding groove of the MHC gene and hence it has achieved much importance for disease resistance research. In goats, there is some documentation of polymorphism at this gene and on the similar lines it was found to be polymorphic in Rohilkhandi goats by PCR-RFLP technique using TaqI and BsaI restriction enzymes. Sequence comparison of two alleles of DRB1 gene of Rohilkhandi goat with other sheep and goat breed assigned Rohilkhandi goat in separate cluster and was also found to be closer to Garole and Scottish blackface sheep breeds. These breeds, Garole and Scottish blackface sheep are documented for their tolerance to gastrointestinal nematodes hence this region can be studied for association study between indicator traits of gastrointestinal nematodiasis and different genotypes at the reported SNPs in Rohilkhandi and other Indian goat breeds.

## Authors’ Contributions

PK, BB, and DS planned and designed the study, KS conducted the study, NRS, BHM, AN, and AP contributed in sample collection, AK, AmK, and MFK helped in analysis of data. All authors have read and approved the final manuscript.

## References

[1] Anonymous (2012). 19^th^ Livestock Census of India.

[2] Aziz M.A. (2010). Present status of the world goat populations and their productivity. Lohmann Inf.

[3] Dukkipati V.S.R., Blair H.T., Garrick D.J., Murray A. (2006). Ovar-Mhc’- Ovine major histocompatibility complex: Role in genetic resistance to diseases. N. Z. Vet. J.

[4] Yakubu A., Salako A., De Donato M., Takeet M., Peters S., Adefenwa M., Okpeku M., Wheto M., Agaviezor B., Sanni T., Ajayi O., Onasanya G., Ekundayo O., Ilori B., Amusan S., Imumorin I. (2013). Genetic diversity in exon 2 of the major histocompatibility complex class II DQB1 locus in Nigerian goats. Biochem. Genet.

[5] Tizard I.R. (2004). Veterinary Immunology.

[6] Garrigan D., Hedrick P.W. (2003). Perspective: Detecting adaptive molecular polymorphism: Lessons from the MHC. Evolution.

[7] Santucci F., Ibrahim K.M., Bruzzone A., Hewit G.M. (2007). Selection on MHC-linked microsatellite loci in sheep populations. Heredity.

[8] Brujeni G.H.N., Emam M., Mahmoudzadeh H., Hamedmonfared E., Talebnia Jahromi R., Rezaei H. (2009). Typing of ovar-DRB1 second exon with PCR-RFLP technique in Iranian Shaul Sheep. Iran. J. Vet. Res.

[9] Jamshidi R., Brujeni G.H.N., Derakhshandeh A., Talebnia R. (2011). Exon 2 Ovar-DRB1 gene polymorphism in the Iranian Sangsari sheep. Int. J. Vet. Res.

[10] Sambrook J., Fritsch E.F., Maniatis E.F., Hrsg H. (1989). Molecular Cloning - A Laboratory Manual.

[11] Amills M., Francino O., Sanchez A. (1990). A PCR-RFLP typing method for the caprine MHC class II DRB gene. Vet. Immunol. Immunopathol.

[12] Wright S. (1978). Evolution and the Genetics of Population, Variability Within and Among Natural Populations.

[13] Sanger F., Nicklen S., Coulson A.R. (1997). DNA sequencing with chain terminating inhibitors. Proc. Natl. Acad. Sci. USA.

[14] Karrow N.A., Goliboski K., Stonos N., Schenkel F., Peregrine A. (2014). Review: Genetics of helminth resistance in sheep. Can. J. Anim. Sci.

[15] Gruszczynska J., Brokowska K., Charon K.M., Swiderek W.P. (2004). Restriction fragment length polymorphism of exon 2 Ovar-DRB1 gene in polish heath sheep and polish lowland sheep. J. Appl. Genet.

[16] Li R.Y., Hui W.Q., Jia B., Shi G.Q., Zhao Z.S., Shen H., Peng Q., Lv L.M., Zhou Q.W., Li H.T. (2011). The relationship between MHC-DRB1 gene second exon polymorphism and hydatidosis resistance of Chinese merino (Sinkiang Junken type), Kazakh and Duolang sheep. Parasite.

[17] Nimbkar C., Ghalsasi P.M., Swan A.A., Walkden-Brown S.W., Kahn L.P. (2003). Evaluation of growth rates and resistance to nematodes of Deccani and Bannur lambs and their crosses with Garole. Anim. Sci.

[18] Riggio V., Matika O., Pong-Wong R., Stear M.J., Bishop S.C. (2013). Genome-wide association and regional heritability mapping to identify loci underlying variation in nematode resistance and body weight in Scottish Blackface lambs. Heredity.

